# Latent profile analysis of medication literacy among older adults with chronic comorbidities and Its relationship with technophobia and selfperceived burden

**DOI:** 10.3389/fpubh.2026.1789005

**Published:** 2026-06-03

**Authors:** Qiqi Chen, Junxia Gao, Yali Yan, Sachu La

**Affiliations:** 1 General Surgery Department, The Fifth People’s Hospital of Ningxia Hui Autonomous Region, Shizuishan, China; 2 Foundational and Translational Medical Research Center, Hohhot First Hospital, Hohhot, China; 3 Abnormal (Anaphylactic) Reaction Department, Hohhot First Hospital, Hohhot, China

**Keywords:** chronic co-morbidities, latent profile analysis, medication literacy, self-perceived burden, technophobia

## Abstract

**Background:**

With the ever-increasing globalizing of aging, chronic comorbidity has become both common among the old population. The senile comorbidities pose as notable challenges of escalation of medication and medical expenditures, and loss in the quality of life of the mature patients. The increase in medication literacy would help reduce the land of unsafe drug administration, unfavorable feelings, and also improve the treatment rates among persons. But there is variation in the rate of medication literacy among the older adults living with varying comorbidities of chronic diseases. Thus, this paper utilizes latent profile analysis to segment medication literacy in this population group in an attempt to clarify the features that accompany medication Literacy among the older adults who with the presence of chronic comorbidities. Additionally, it discusses factors that contribute to medication literacy when using different types of chronic conditions, hence developing theoretical underpinnings to the upcoming individualized medication literacy interventions programs as per older adults patients with chronic comorbidities.

**Methods:**

The study is a cross-sectional study of 611 hospitalized patients over the age of 60 years with chronic comorbidities through Grade III hospitals in Shizuishan City in the period between January, 2024 and March, 2024 using the convenience sampling method. The General Data Scale, the Medication Literacy Scale among the Elderly Patients with Chronic Diseases, the Self-perceived Burden Scale and the Technophobia Scale were used to collect information.

**Findings:**

Latent profile analysis (LPA) disclosed that medication literacy of the older adults patients with chronic diseases could be classified into four different groups namely; high medication literacy (17.02%), medication Literacy-low critical type (38.13%), medication Literacy-high critical type (31.26%), and low medication Literacy (13.58%). Influential factor analysis showed that drinking history, educational level, marital status, occupational status, personal monthly income, family location, caregiver involvement, living style, type of medical insurance, daily exercise time, time duration of disease, number of hospitalizations in the past year, personal view of sleep status, age, and self-perceived burden, technophobia, had significant impact among the varied category of chronic disease patients in terms of medication literacy (*p* < 0.05).

**Conclusion:**

Medication literacy among chronic comorbidity patients is largely heterogeneous. It is advised that clinicians should do more specific interventional programs based on the nature of different levels of medication literacy to achieve better medication literacy rates within this category of population to improve treatment effects.

## Introduction

The high pace of aging has resulted in a more pronounced global prevalence of chronic diseases, therefore becoming a major global issue to the public health of the world. The findings of the World Health Organization indicate that the number of people who die due to chronic diseases Fitzal per annum is 40.1 million, which is 74.0 per cent of all deaths worldwide (1). Chronic co-morbidities are associated with concurrent presence of two or more chronic conditions which may pose negative effects to the daily life of an individual especially where a large number of co-morbidities are present (2). Co-morbidities of chronic diseases are common among the geriatric population in China with 76.5% of the geriatric population in China having two or more chronic illnesses (3). Factors influencing these co-morbidities include the distance from residence to major roads (4), age (5), income, and mental health (6). In India, among adults aged 45 years and older, the incidence of chronic co-morbidities is as high as 25%, with approximately 30% of these individuals experiencing psychiatric problems, thereby increasing the demand for local healthcare services (7). A survey of 2,426 researchers in the United States found that the incidence of shared co-morbidity was 80.2%, with age, smoking, physical activity level, and education level identified as influencing factors (8). Medication literacy is the capability of an individual to acquire, comprehend, communicate, compute, and process concrete information on drugs, to make sound drug treatment and health-based choices, as well as to take medicine safely and efficiently (9). Studies have found that factors such as disease duration, number of oral medications (10, 11), care environment (12), income level, occupational status (13), medical insurance payment methods, and medication label retention (14) significantly influence patients ‘medication literacy. A survey of 362 older adults hypertensive patients revealed a significant positive correlation between social support and medicationliteracy levels, with patients receiving higher social support more inclined to actively share and exchange medication experiences (15). Increasing knowledge of medications helps patients better manage their treatment and improves the effectiveness of the treatment. Improving patients’ knowledge of medications can also reduce errors and adverse effects of medication, expanding its therapeutic potential and indirectly improving mental health (16). On the other hand, lower levels of medication literacy can cause or exacerbate anxiety and depression, adversely affecting patients’ beliefs and attitudes toward the use of medicines (17). The study also showed that there is a correlation of anxiety and depression with the patients’ perceived burden, which indicates that perceived burden can cause or worsen psychological stress and may also reduce the quality of life.

There is a two-way link involving mental health and understanding of medications. Poor understanding of medications may lead to medication insecurity and consequently increases mental health issues. Conversely, mental health issues may prevent individuals from understanding and using medication, creating a negative cycle. Social support can mitigate this negative cycle, and can transform the perception of a burden through positive health behavioral changes. Negative cycle impacts can be lessened through the provision of emotional sustenance, information, and practical support. Therefore, when designing and implementing interventions, it is essential to integrate knowledge education, psychological support, and social resources to multi dimensionally enhance medication knowledge levels and overall quality of life in older adults patients with chronic comorbidities.

Currently, most studies on medication literacy among older adults patients with chronic comorbidities concentrate on assessing its overall level and identifying influencing factors. However, many of these investigations employ traditional data analysis methods, such as regression analysis (10, 18, 19), which overlook the multidimensional characteristics of medication literacy and the heterogeneity within samples. This limitation may hinder the effectiveness of future targeted intervention studies for older adults patients with chronic diseases. Different chronic conditions in the older adults population exhibit varying levels of medication Literacy. Consequently, we classified and analyzed the heterogeneity of medication literacy among older adults patients with chronic comorbidities to elucidate the characteristics and influencing factors associated with different groups, thereby facilitating the development of targeted intervention programs in future research. The latent profile analysis employed by Lanxin et al. (20) classified self-management levels of older adults with comorbidities into three separate groups, namely, good self-management (19.4%), medium self-management (27.9%), and low self-management (52.7). This classification emphasizes that the older adults are not homogeneously self-managed and thus conducting a specific intervention research at a later stage is important.

Therefore, the aim of this study is to classify the medication literacy of older adults patients with chronic disease comorbidities through latent profile analysis (LPA) and to analyze the influencing factors. Meanwhile, the correlation analysis of medication literacy dimensions with self-perceived burden and technophobia in older adults patients with comorbid chronic diseases was conducted. This approach aims to provide a reference for developing stratified intervention programs, particularly for individuals with low medication literacy, ultimately enhancing the medication literacy levels within this population.

## Methods

### Study design and participants

This study employed convenience sampling to select patients with chronic comorbidities hospitalized at a tertiary general hospital in Shizuishan City from January to April 2024 as the study subjects. Inclusion criteria included: patients aged ≥60 years, in a hospitalized state, and with a diagnosis of two or more chronic diseases confirmed by the attending physician in the admission diagnosis certificate. The chronic diseases involved included, but were not limited to, tumors, hypertension, diabetes, coronary heart disease, cerebrovascular diseases, hyperlipidemia, hyperuricemia, asthma, chronic obstructive pulmonary disease (COPD), gastroesophageal reflux disease (GERD), atrial fibrillation, thyroid diseases, and arthritis. Exclusion criteria encompassed: those who currently are in another clinical trial or study, and those that have temporarily dropped out of the study due to an abrupt illness or other causes. This study has been approved by the Ethics Committee of the Fifth People’s Hospital of Ningxia Hui Autonomous Region (Approval No.: NXWYLLL-2023-0024). The sample size was determined based on the sample size calculation formula for cross-sectional surveys (21), taking into account the study objectives, expected effect size, significance level, and statistical power to ensure the representativeness and reliability of the study results.

The general data for this study included 17 independent variables, the Chinese version of the medication Literacy Assessment Scale included 4 independent variables, the Chinese version of the Self-Perceived Burden Scale included 1 independent variable, and the Chinese version of the Technical Anxiety Scale included 3 independent variables, resulting in a total of 25 independent variables. Therefore, the formula for calculating the sample size is N = (17 + 4 + 1 + 3) * 10 = 250. Considering an expected invalid questionnaire rate of 20%, the final sample size was calculated as N = 250/(1–20%) ≈ 313. In this study, a total of 632 questionnaires were distributed, and 21 invalid questionnaires were excluded (17 with Linear response, where the research subject selects the same answer option for all or most questions during questionnaire completion, e.g., selecting “strongly agree” for all items or assigning “3 points” to all items, fails to demonstrate the expected variability and thought process. This response pattern is typically considered invalid or arbitrary and 4 with contradictory responses). Consequently, a total of 611 valid questionnaires were recovered, yielding an effective recovery rate of 96.68%.

### Instruments

#### General information questionnaire

To ensure that the collected baseline data comprehensively reflects the demographic characteristics of the study subjects, this study conducted systematic literature reviews in relevant fields both domestically and internationally during the questionnaire design process. We systematically organized commonly used sociodemographic and health-related factor indicators, and based on this, consulted multiple experts with experience in clinical and public health research to conduct multiple rounds of discussions and optimization on item design. Finally, overall demographic picture was created which included 17 items, with some specific content material being: gender, body mass index (BMI), smoking history, alcohol consumption history, level of education, marital status, occupational status, personal monthly income, household address, primary caregiver, lifestyle, type of medical insurance, amount of exercise time taking every day, duration of disease, number of hospitalizations in the last year, self-rated sleep status and age.

The questionnaire plan does not only support gathering the simple demographic variables, but also captures multi-layered variables that may affect the health behavior and medical requirements of the older adults individuals with chronic illnesses, which will present a full-fledged data base to be used later to undertake the stratified data analysis, correlation analysis, and multivariate model building.

#### Medication literacy scale for older adults patients with chronic diseases

This study used the Medication Literacy Scale of older adults patients with chronic disease (22). This scale has 23 items in four dimensions as the ability to acquire information, medication knowledge reserve, communication and interaction skills, and critical thinking ability. The 0.956, 0.945, 0.876, and 0.942 coefficients of the Cronbach *α* of each dimension reveal that the internal consistency is good. Cronbachs 0.958 is the overall Cronbach coefficient which reveals high reliability in the scale. Confirmatory factor analysis was used to perform a validity analysis that showed that model fit was significant. The items are rated using a 5-point Likert (1 = never, 5 = always) scale. The scores will be higher in showing more medication knowledge by older adults patients with chronic diseases. The *α* coefficient of the scale used in this study was 0.956.

#### Self-perceived burden scale

In this research, the instrument that was utilized was the Self-Perceived Burden Scale (SPBS) (23), which is a special instrument that was used to measure the subjective burden of patients with chronic illnesses in their day-to-day life and unpaid care. The scale will consist of 10 questions assigned a 5 point Likert scale (1 = never, 5 = always), with a maximum total score of 0–50 points. In the scoring criteria, the higher the score, the higher the perceived burden, and so the lowest score (0–25) implies low burden, moderate burden (26–33), and high burden (34–50). There have been previous successes of the SPBS on reliability and validity in various cross-cultural and national studies where the coefficient of Cronbach *α* of the original scale stands at 0.85. The α coefficient in the sample of this research was elevated to 0.897, which once again justified its internal consistency and stability in the subjects of the research at hand. Moreover, the scale items are also brief and understandable, which is effective as respondent bias is minimized and the scale forms a credible foundation on which to base measurement of Self-perceived burden of patients with chronic diseases.

#### Technophobia scale

The Teenophobia Scale (24) in this study would be used to determine the feeling of anxiety regarding technology among the older adults. The scale is made up of 13 questions that are grouped into three dimensions which include technology stress, technology fear and privacy and security concerns. The rating of each item is based on the 5-point Likert (1 = completely inconsistent, 5 = completely consistent), where a higher combination of the total scores creates a higher result of technological anxiety. The value of the Cronbachs alpha coefficient of the entire scale is 0.867, whereas in this research Cronbachs alpha coefficient of this very scale was 0.932.

#### Data collection and quality control methods

This study was conducted using electronic questionnaires to perform a cross-sectional survey in a tertiary Grade A hospital in the city of Shizuishan. Before the electronic survey, permits and support from the hospital management and related department heads were sought to ease the operation of the survey. Using the WeChat workgroups to distribute links and QR codes of the questionnaires, people in the target population were reached and surveyed. The questionnaires were preceded by a page of instructions, to explain the objectives and significance of the study, and the instructions of how to fill it out, to simplify the survey and inform the raters. Nurses were instructed to return the questionnaires after filling them out from their own consent. Responses were collected from raters through a survey link to guarantee their anonymity. These steps were taken to preserve the quality of the data. To minimize response bias, rigorous quality control procedures were adopted. We ensured anonymity and confidentiality by refraining from collecting personally identifiable information—such as names or addresses—and clearly communicating the study’s objectives to participants. These measures were designed to prevent social desirability bias. Second, we conducted validity tests to refine the measurement instruments and clarify conceptual definitions prior to implementation, thereby reducing measurement bias and enhancing data accuracy. Third, to ensure standardization, all investigators received uniform training on the use of standardized interview protocols and written manuals. Finally, a double-entry verification procedure was implemented during data processing, whereby two independent researchers entered and cross-checked the data. This rigorous mechanism ensured high data reliability and strengthened the internal validity of the findings.

### Statistical methods

This study employs Mplus 8.3 in constructing the possible profile model of the medication literacy of geriatric patients confronted with persistent health challenges. The four dimensions of the Chinese version of the medication literacy framework in older adults with chronic conditions were chosen to be the explicit indicators. A 5-profile approach was utilized to analyze the implicit indicators. Most of the evaluative indicators were based on Akaike Information Criterion (AIC), Bayesian Information Criterion (BIC), adjusted BIC (aBIC), and Entropy—the higher the AIC, BIC and aBIC, the worse the model fit, while the Entropy value, which falls between 0 and 1, is the better the model fit. The AIC and BIC of the LMRT and BLRT tests were of the adjusted likelihood ratio tests, which measured the correlation between the two K models and the K-1 models, where a *p* value of less than 0.05 indicated that the K model was superior to the K-1 model (25). Once the ideal latent category model was determined, the SPSS 25.0 software was used to undertake further data analysis and processing. The data were found to follow a normal distribution; therefore, descriptive statistics were presented as mean ± standard deviation (mean ± SD), while categorical data were expressed as counts and relative frequencies (%). To further investigate the factors influencing the distribution of drug literacy latent categories, this study employed the latent categories as the dependent variable and included variables with statistically significant results from the univariate analysis as independent variables in a multivariate ordered logistic regression model, with a significance level set at *α* = 0.05. For intergroup comparisons, chi-square tests (*χ*^2^ test) were used for categorical data, and the nonparametric Kruskal–Wallis H test was applied to quantitative data, with *p* < 0.05 regarded as statistically significant. In this study, the Harman single - factor test method was employed to calculate and test all variables. The results indicate that there are four factors with eigenvalues greater than 1. The first principal component obtained before rotation accounts for 44.712% of the total factor loading, which does not exceed the critical value of 50%. This suggests that there is no severe common method bias in the questionnaire survey data of this study.

## Results

### General demographic information and medication literacy status of chronic disease comorbidities in the older adults

This study included a total of 611 patients with chronic diseases, with an average age of 69.89 ± 7.72 years, comprising 339 males and 272 females. The body mass index (BMI) distribution was as follows: 62 patients had a BMI <18.5, 276 patients had a BMI in the range of 18.5 to <24, and 273 patients had a BMI ≥24. Among the participants, there were 219 former smokers and 392 non-smokers, as well as 232 previous drinkers and 379 abstainers. Regarding educational background, 57 individuals were illiterate, 172 had completed primary school, 186 had junior high school education, 145 had secondary school or junior college education, and 51 had attained a bachelor’s degree or higher. The general demographic characteristics of other older adults patients with chronic diseases are presented in Table 1. The scores for the four dimensions of medication literacy among older adults patients with chronic diseases were 16.31 ± 5.45, 20.25 ± 6.28, 16.90 ± 5.18, and 23.76 ± 7.12, with a cumulative score of 77.29 ± 22.78. The self-perceived burden score was 28.11 ± 8.81, and the technical anxiety score was 28.85 ± 12.50.

**Table 1:** General demographic information of older adults patients with chronic disease comorbidity.

Variable	*n* (%)	Variable	*n* (%)
Gender		Caregivers	
Male	339 (55.5)	Spouse	332 (54.3)
Female	272 (44.5)	Children	217 (35.5)
BMI		Others	45 (7.4)
<18.5	62 (10.1)	Relatives	17 (2.8)
18.5 ≤ X<24	276 (45.2)	Residential mode	
≥24	273 (44.7)	Living with husband and wife	396 (64.8)
Smoking history		Living alone	62 (10.1)
Yes	219 (35.8)	Children living together	135 (22.1)
No	392 (64.2)	Other	18 (2.9)
Drinking history		Medical insurance type	
Yes	232 (38.0)	Employee medical insurance	334 (54.7)
No	379 (62.0)	Resident medical insurance	256 (41.9)
Degree of education		Self-financing	21 (3.4)
Primary school	172 (28.2)	Daily exercise duration	
Junior high school	186 (30.4)	Less than 30 min	318 (52.0)
Vocational or junior college	145 (23.7)	30 min to 1 h	196 (32.1)
Bachelor’s degree or above	51 (8.3)	1–2 h	71 (11.6)
Not attending school	57 (9.3)	More than 2 h	26 (4.3)
Marital status		Course of disease	
Married	510 (83.5)	Less than 6 months	191 (31.3)
Unmarried	31 (5.1)	6 months −1 year	108 (17.7)
Widowed or widowed	54 (8.8)	1–3 years	126 (20.6)
Divorce	16 (2.6)	3–5 years	86 (14.1)
Status of occupational		5 years and above	100 (16.4)
On the job	157 (25.7)	Number of hospitalizations in the past year	
Retired	384 (62.8)	0 times	120 (19.6)
Unemployment	70 (11.5)	1–2 times	307 (50.2)
Personal monthly income		3–5 times	124 (20.3)
Below 3,000 CNY	222 (36.3)	5 times or more	60 (9.8)
3,000–5,000 CNY	305 (49.9)	Self-rated sleep status	
5,000–10,000 CNY	79 (12.9)	Very good	155 (25.4)
Above 10000 CNY	5 (0.8)	General	348 (57.0)
Family location		Relatively poor	108 (17.7)
City	497 (81.3)		
Rural areas	114 (18.7)		

### Results of potential profling of medication literacy in older adults patients with chronic disease comorbidity

In this study, we analyzed a total of five models concerning the scores of four dimensions of medication literacy among patients with chronic comorbidities, with the analysis indicators for each model presented in Table 2. As the number of potential profiling models increases, the Akaike Information Criterion (AIC), Bayesian Information Criterion (BIC), and Adjusted Bayesian Information Criterion (ABIC) decrease, while Entropy increases, This study selected four types of models as the optimal potential analysis models. There are 3 reasons why is model 4 better than model 5: First, Although Model 5 exhibits a higher entropy value (Entropy = 0.931) than Model 4 (Entropy = 0.920), indicating relatively higher classification accuracy, both Model 4 and Model 5 show *p*-values < 0.001 in LMRT and BLRT tests. This suggests that statistically, Model 5 does not provide sufficient evidence to outperform Model 4. Second, From the perspective of categorical probability, Model 4 demonstrates a more rational probability distribution with clearer distinctions between categories. In contrast, Model 5 exhibits some low-probability classifications (e.g., 0.062, calculated as 611 × 0.062 ≈ 37.88), which fails to meet the minimum sample size requirement of 50 for the profile analysis grouping. This finding indirectly validates the rationale for selecting the four-category model. Third, the Logistic Regression Analysis results demonstrated that when the classification outcome was Model 4, the 95% confidence interval did not cross the null line 1, indicating statistically significant findings in this study. Therefore, the four-category model is preferable to the five-category model. Consequently, based on model fit indices and practical significance, this study selected four types of models as the optimal potential analysis models for medication literacy in older adults patients with chronic comorbidities.

**Table 2:** Model indicators of potential medication literacy in older adults patients with chronic disease comorbidity.

Model	AIC	BIC	aBIC	Entropy	*p*		Categorical probability
					LMR	BLRT	
1	15678.281	15713.602	15688.203	—	—	—	1.000
2	14319.546	14376.942	14335.670	0.875	*p*<0.001	*p*<0.001	0.531/0.468
3	13516.790	13596.262	13539.116	0.915	*p*<0.001	*p*<0.001	0.178/0.458/0.363
4	13050.919	13152.466	13079.446	0.920	*p*<0.001	*p*<0.001	0.381/0.135/0.170/0.312
5	12725.109	12848.732	12759.838	0.931	*p*<0.001	*p*<0.001	0.175/0.062/0.331/0.268/0.163

**Figure 1:**
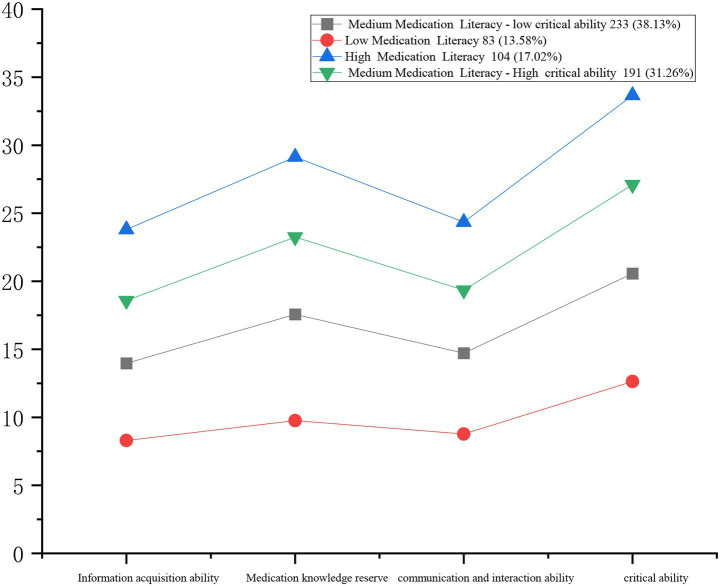
Distribution of four potential characteristic characteristics of medication literacy in older adults patients with chronic disease comorbidity.

Following the determination of the potential category model, the potential profiles of the four categories across the four dimensions of medication literacy were obtained. (see Figure 1). Class 1 comprised 233 patients (38.13%) and was designated as Medium Medication Literacy-Low Critical Ability due to their medium level of overall medication literacy and low score in the critical ability dimension. The second category included 83 patients (13.58%) with the lowest overall level of Medication Literacy and was labeled Low Medication Literacy. Category 3 encompassed 104 patients (17.02%) who exhibited the highest level of overall Medication Literacy and was thus named High Medication Literacy. Class 4 consisted of 191 patients (31.26%) and was referred to as Medium Medication Literacy-High Critical Ability, characterized by a medium level of overall medication literacy but a high score in the critical ability dimension.

### Correlation analysis of the variables

The correlation coefficients of the main variables in this study are presented in Table 3. The results demonstrated highly significant pairwise correlations (correlation coefficients ranging from 0.810 to 0.900) among the first four competency variables (Information acquisition ability, Medication knowledge reserve, Communication and interaction skills, Critical thinking ability), indicating strong interrelatedness among these competencies in the study subjects. In contrast, Self-perceived burden showed.

**Table 3:** The correlation coefcients of the main variables (*n* = 611).

Number	Items	Information acquisition ability	Medication knowledge reserve	Communication and interaction skills	Critical thinking ability	Self-perceived burden	Technophobia
1	Information acquisition ability	1					
2	Medication knowledge reserve	0.861**	1				
3	Communication and interaction skills	0.819**	0.896**	1			
4	Critical thinking ability.	0.810**	0.881**	0.900**	1		
5	Self-perceived burden	0.167**	0.163**	0.177**	0.186**	1	
6	Technophobia	0.242**	0.235**	0.225**	0.246**	0.550**	1

The correlation coefficients with the first four abilities were relatively low (0.163–0.186), indicating limited association between Self-perceived Burden and ability levels. Technophobia showed slightly higher correlation coefficients with the first four abilities (0.225–0.246) and exhibited the strongest correlation with Self-perceived Burden (0.550), suggesting a moderate positive correlation between Technophobia and Self-perceived Burden.

### Univariate analysis of potential medication literacy profiles of older comorbid patients

Through a univariate analysis of medication literacy in older adults patients with chronic comorbidities, this study identified several important influencing factors across four potential categories. These factors include drinking history, educational level, marital status, occupational status, personal monthly income, family location, caregiver support, lifestyle, type of medical insurance, daily exercise duration, course of disease, number of hospitalizations in the past year, self-rated sleep status, age, self-perceived burden, and technical anxiety. The analysis revealed statistically significant differences (*p* < 0.05), as illustrated in Table 4.

**Table 4:** Univariate analysis of the four potential categories of medication literacy in older adults patients with chronic disease comorbidity.

Variable	C1	C2	C3	C4	$\chi^{2}$ /*F*	*p*
Gender					$\chi^{2}$ =6.791	0.079
Male	126 (54.08)	37 (44.58)	65 (62.5)	111 (57.81)		
Female	107 (54.92)	46 (55.42)	39 (37.5)	80 (41.88)		
BMI					$\chi^{2}$ =9.426	0.151
<18.5	25 (10.72)	13 (15.67)	4 (3.84)	20 (10.47)		
18.5 ≤ X<24	103 (44.21)	32 (38.55)	56 (53.84)	85 (44.50)		
≥24	105 (45.06)	38 (45.78)	44 (42.30)	86 (45.02)		
Smoking history					$\chi^{2}$ =7.750	0.051
Yes	85 (36.48)	19 (22.89)	43 (41.34)	72 (37.69)		
No	148 (63.51)	64 (77.11)	61 (58.66)	119 (62.30)		
Drinking history					$\chi^{2}$ =21.234	<0.001
Yes	78 (33.47)	18 (21.69)	53 (50.96)	83 (43.45)		
No	155 (66.53)	65 (78.31)	51 (49.04)	108 (56.54)		
Degree of education					$\chi^{2}$ =89.908	
Primary school	70 (30.04)	37 (44.58)	16 (15.38)	49 (25.65)		<0.001
Junior high school	73 (31.33)	17 (20.48)	26 (25.00)	70 (36.64)		
Vocational or junior college	47 (20.17)	7 (8.43)	41 (39.42)	50 (26.17)		
Bachelor’s degree or above	13 (5.58)	4 (4.82)	20 (19.23)	14 (7.33)		
Not attending school	30 (12.87)	18 (21.69)	1 (0.96)	8 (4.19)		
Marital status					$\chi^{2}$ =28.245	<0.001
Married	191 (81.97)	59 (71.08)	95 (91.34)	165 (86.38)		
Unmarried	7 (3.00)	8 (9.64)	6 (5.77)	10 (5.24)		
Widowed or widowed	26 (11.16)	15 (18.07)	2 (1.92)	11 (5.76)		
Divorce	9 (3.87)	1 (0.12)	1 (0.96)	5 (2.62)		
Status of occupational					$\chi^{2}$ =65.788	<0.001
On the job	42 (18.03)	13 (15.67)	57 (54.80)	45 (23.56)		
Retired	166 (71.24)	52 (62.65)	41 (39.42)	125 (65.45)		
Unemployment	25 (10.73)	18 (21.69)	6 (5.76)	21 (10.99)		
Personal monthly income					$\chi^{2}$ =80.260	<0.001
Below 3,000 CNY	92 (39.48)	51 (61.45)	18 (17.30)	61 (31.93)		
3,000–5,000 CNY	118 (50.64)	28 (33.73)	49 (47.11)	110 (57.59)		
5,000–10,000 CNY	21 (9.01)	3 (3.61)	35 (33.65)	20 (10.47)		
Above 10,000 CNY	2 (0.85)	1 (1.20)	2 (1.92)	0 (0)		
Family location					$\chi^{2}$ =15.877	<0.001
City	191 (81.97)	58 (69.88)	96 (92.30)	152 (79.58)		
Rural areas	42 (18.03)	25 (30.12)	8 (7.69)	39 (20.42)		
Caregivers					$\chi^{2}$ =41.072	<0.001
Spouse	120 (51.50)	29 (34.94)	74 (71.15)	109 (56.06)		
Children	93 (39.91)	47 (56.63)	16 (15.38)	61 (31.94)		
Others	13 (5.58)	6 (7.22)	12 (11.53)	14 (7.33)		
Relatives	7 (3.00)	1 (1.2)	2 (1.92)	7 (3.66)		
Residential mode					$\chi^{2}$ =41.387	<0.001
Living with husband and wife	147 (63.09)	32 (38.55)	82 (78.84)	135 (70.68)		
Living alone	22 (9.44)	15 (18.07)	10 (9.61)	15 (7.85)		
Children living together	57 (24.46)	33 (39.76)	11 (10.57)	34 (17.80)		
Other	7 (3.00)	3 (3.61)	1 (0.96)	7 (3.66)		
Medical insurance type					$\chi^{2}$ =18.891	0.004
Employee medical insurance	125 (53.65)	31 (37.35)	62 (59.61)	116 (60.73)		
Resident medical insurance	100 (42.92)	48 (57.83)	42 (40.38)	66 (34.55)		
Self-financing	8 (3.43)	4 (4.82)	0 (0)	9 (4.71)		
Daily exercise duration					$\chi^{2}$ =26.159	0.002
Less than 30 min	116 (49.79)	57 (68.67)	62 (59.61)	83 (43.45)		
30 min − 1 h	84 (36.05)	15 (18.07)	23 (22.11)	74 (38.74)		
1–2 h	26 (11.16)	6 (7.22)	12 (11.53)	27 (14.13)		
More than 2 h	7 (3.00)	5 (6.02)	7 (6.73)	7 (3.66)		
Course of disease					$\chi^{2}$ =64.311	<0.001
Less than 6 months	57 (24.46)	19 (22.89)	52 (50.00)	63 (32.98)		
6 months to 1 year	49 (21.03)	10 (12.05)	17 (16.34)	32 (16.75)		
1–3 years	54 (23.18)	13 (15.67)	22 (21.15)	37 (19.37)		
3–5 years	42 (18.03)	11 (13.25)	12 (11.53)	21 (10.99)		
5 years and above	31 (13.30)	30 (36.14)	1 (0.96)	38 (19.89)		
Number of hospitalizations in the past year					$\chi^{2}$ =50.260	<0.001
0 times	29 (12.45)	13 (15.67)	38 (36.53)	40 (20.94)		
1–2 times	123 (52.79)	41 (49.40)	55 (52.88)	88 (46.07)		
3–5 times	64 (27.46)	20 (24.10)	5 (4.80)	35 (18.32)		
5 times or more	17 (7.29)	9 (10.83)	6 (5.76)	28 (14.66)		
Self-rated sleep status					$\chi^{2}$ =19.160	<0.001
Very good	41 (17.59)	11 (13.25)	55 (52.88)	48 (25.13)		
General	146 (62.66)	48 (57.83)	43 (41.34)	111 (58.12)		
Relatively poor	46 (19.74)	24 (28.92)	6 (5.76)	32 (16.75)		
Age	69.92 ± 7.36	75.72 ± 9.19	66.50 ± 5.13	69.11 ± 7.27	*F* = 25.939	<0.001
self-perceived burden	27.65 ± 7.07	25.35 ± 7.96	30.89 ± 12.71	28.35 ± 8.09	*F* = 6.600	<0.001
Technophobia	27.99 ± 10.78	22.73 ± 10.83	33.12 ± 16.87	30.25 ± 11.21	*F* = 12.490	<0.001

C1: medium medication literacy - low critical ability 233 (38.13%), C2: low medication literacy 83 (13.58%), C3: high-medication literacy 104 (17.02%), C4: medium medication literacy-high critical ability 191 (31.26%).

### Multi factorial analysis of potential categories of older co-morbid patients

A disordered multi-classification logistic regression model was constructed with four categories of medication literacy among older adults patients with chronic disease comorbidities as dependent variables, and statistically significant factors identified in univariate analysis as independent variables. The Medium Medication Literacy-low critical ability group served as the reference group. The multivariate logistic regression analysis revealed that technical anxiety, marital status, daily exercise duration, disease duration, self-perceived burden, educational level, annual hospitalization frequency, self-rated sleep status, and personal monthly income were significant influencing factors of medication literacy in older adults patients with chronic disease comorbidities (*p* < 0.05). Specifically, compared to the Medium Medication Literacy-low critical ability group, those exhibiting technical anxiety, being unmarried, and engaging in 30 min to 1 h of daily exercise, as well as having chronic comorbidities lasting 6 months to 5 years, were more likely to be classified as having Low Medication Literacy. Conversely, compared to the Medium Medication Literacy -low critical ability group, older adults patients with chronic disease comorbidities who reported a self-perceived burden, possessed an education level ranging from junior high school to bachelor’s degree or above, engaged in daily exercise for 30 min to 1 h, had a disease duration of less than 6 months to 5 years, experienced 3–5 hospitalizations per year, and rated their sleep status as normal or very good were more likely to be classified as having High Medication Literacy. Lastly, older adults patients with chronic comorbidities characterized by technical anxiety, an education level from junior high school to bachelor’s degree or above, a monthly personal income of less than 3,000 to 5,000 yuan, and 1–5 hospitalizations per year were more likely to be classified as having Medium Medication Literacy - High critical ability, as illustrated in Table 5.

**Table 5:** Logistic regression analysis of medication literacy in older adults patients with chronic disease comorbidity - with medium medication literacy-low critical ability as the reference group (*n* = 611).

Category variable		*β*	SE	Wald *χ*^2^	*p*	OR	95% CI	
C2	Intercept	−5.578	2.225	6.285	0.012			
	Age	0.093	0.022	17.316	0.000	1.098	1.051	1.147
	self-perceived burden	−0.017	0.022	0.579	0.047	0.983	0.942	1.027
	Technophobia	−0.046	0.016	8.285	0.004	0.955	0.926	0.986
	Vocational or junior college	0^a^	—	—	—	—	—	—
	Married	1.994	0.854	5.454	0.020	7.346	1.378	39.161
	Unmarried	0^a^	—	—	—	—	—	—
	Daily exercise Less than 30 min	−1.035	0.518	3.999	0.046	0.355	0.129	0.980
	Daily exercise 30 min to 1 h	0^a^	—	—	—	—	—	—
	Course of disease less than 6 months	−1.091	0.506	4.649	0.031	0.336	0.125	0.906
	Course of disease 6 months to 1 year	−1.759	0.518	11.521	0.001	0.172	0.062	0.476
	Course of disease 1–3 years	−1.266	0.483	6.855	0.009	0.282	0.109	0.727
	Course of disease 3–5 years	−1.774	0.523	11.515	0.001	0.170	0.061	0.473
	Course of disease more than 5 years	0^a^	—	—	—	—	—	—
C3	Intercept	−23.973	**2.706**	78.457	0.000			
	self-perceived burden	0.054	0.020	7.170	0.007	1.055	1.015	1.098
	Below 3,000 CNY	−1.762	0.503	12.275	0.000	0.172	0.064	0.460
	3,000–5,000 CNY	−1.243	0.407	9.339	0.002	0.288	0.130	0.640
	5,000–10,000 CNY	0^a^	—	—	—	—	—	—
	Caregivers children	−1.498	0.656	5.212	0.022	0.224	0.062	0.809
	Caregivers others	0^a^	—	—	—	—	—	—
	Daily exercise less than 30 min	−0.991	0.460	4.627	0.031	0.371	0.151	0.916
	Daily exercise 30 min to 1 h	0^a^	—	—	—	—	—	—
	Number of hospitalizations 0 times	1.377	0.531	6.725	0.010	3.962	1.400	11.214
	Number of hospitalizations more than 3 times	0^a^	—	—	—	—	—	—
	Course of disease less than 6 months	2.306	1.137	4.118	0.042	10.036	1.082	93.100
	Course of disease 6 months to 1 year	2.866	1.142	6.300	0.012	17.559	1.874	164.533
	Course of disease 1–3 years	2.625	1.173	5.007	0.025	13.802	1.385	137.544
	Course of disease 3–5 years	0^a^	—	—	—	—	—	—
	Self-rated sleep status very good	2.249	0.581	15.007	0.000	9.479	3.038	29.577
	Self-rated sleep status general	1.115	0.553	4.067	0.044	3.050	1.032	9.016
	Self-rated sleep status relatively poor	0^a^	—	—	—	—	—	—
C4	Intercept	−1.103	1.526	0.522	0.001			
	Technophobia	0.022	0.010	4.583	0.032	1.022	1.002	1.043
	Course of disease less than 6 months	−0.728	0.359	4.119	0.042	0.483	0.239	0.975
	Course of disease 6 months to 1 year	−0.760	0.348	4.759	0.029	0.468	0.236	0.926
	Course of disease 1–3 years	−0.938	0.381	6.063	0.014	0.391	0.185	0.826
	Course of disease 3–5 years	0^a^	—	—	—	—	—	—

C2: low medication literacy 83 (13.58%), C3: high-medication literacy 104 (17.02%), C4: medium medication literacy-high critical ability 191 (31.26%). ^a^denotes the unstandardized regression coefficient, reflecting the actual change in the outcome per unit increase in the predictor.

## Discussion

The results of this study indicated that the average medication literacy score among older adults patients with chronic disease comorbidities was 77.29 ± 22.78, placing it at a moderate level within this population. The scores across the four dimensions of medication Literacy were as follows: information acquisition ability scored 16.31 ± 5.45, drug knowledge reserve scored 20.25 ± 6.28, communication and interaction ability scored 16.90 ± 5.18, and critical ability scored 23.76 ± 7.12. Notably, the score for information acquisition ability was relatively low. This suggests a need for targeted training to enhance the information acquisition skills of older adults patients, thereby improving their overall medication Literacy.

According to the Latent Profile Analysis (LPA), this research was able to identify the medication Literacy features in four different groups of chronic disease comorbid patients of various ages. The outcomes of both model indexes showed positive results, where the difference is significant in medication literacy level in each of the categories. There are 233 people in category 1, which is Medium Medication Literacy with low critical ability (38.13%). The critical ability of this group is still quite poor and they score medium in each of the dimensions. The category 2 with Low Medication Literacy (13.58) comprises 83 people (13.58) and is the smallest group, as it scored less than other categories in all dimensions. The patients in this group have some level of technophobia and practices less than an hour of daily exercise, which is consistent with the reports of Zhong et al. (26) of the study. Category 3 is named as High Medication Literacy (17.02-percent) which has 104 people who formed a higher score in all the dimensions than in the other categories. This segment is more likely to be more educated, has been hospitalized more in the last 1 year, has been living with the illness, and has a significant level of self perceived burden, which is similar to the findings of the research by Shen et al. (27). Category 4 is Medium Medication literacy High Critical Ability (191 people 31.26%). The patients with such a category have lower monthly incomes, a longer time of the disease, a high level of education, and numerous hospitalizations in the year of which PLAZA-ZAMORA J, L findings are supported (19). As such, nursing personnel need to effectively classify the type of medication literacy in patients, the root cause behind the difference in medication literacy level, and proactively introduce specific interventions to help patients acquire precise drug knowledge and correct their harmful lifestyle habits. Moreover, high-profile medication Literacy patients are advised to share their experience with the patients representing different demographics in order to improve medication literacy among other demographic groups.

The findings of this study indicate that multiple factors influence the medication literacy levels of older adults patients with chronic comorbidities across different categories (*p* < 0.05), which can be categorized into three groups: Economic factors, including monthly income, type of medical insurance, and household address, directly affect patients ‘access to health information and medical resources; Social factors encompass education level, marital status, occupational status, caregiver support, lifestyle, daily physical activity duration, disease duration, hospitalization frequency in the past year, and self-rated sleep status, reflecting the role of social support and living conditions in knowledge acquisition; Psychological factors include self-perceived burden and technology phobia, indicating the impact of patients’ internal psychological experiences on the mastery of medication knowledge.

This study elucidates the underlying logic of the TAM in explaining the mechanisms of pharmacological literacy development. Data demonstrate an exceptionally strong interdependence among four core competencies: Information acquisition ability, Medication knowledge reserve, Communication and Interventions in the study add on the body of knowledge in (13, 26, 28–31) that geriatricians and clinicians ought to apply an inclusive approach that is based on multiple dimensions to improve their patients’ awareness on medication.

Among such influencing factors, family income, type of health insurance, and employment status are of particular concern to medication literacy (13, 28). Patients of low income, who need long term medication supply, do not possess adequate health insurance, resulting in high economic burdens. This, in turn, leads to legally unauthorized drug discontinuation, decreased drug intake, and overall poor medication literacy (29). Furthermore, the research noted that the patients with the highest number of hospitalizations in the previous year had the highest scores in medication literacy, thus affirming that prolonged hospitalization is a notable determinant of medication literacy (26).

In line with the primary findings of this study, existing studies demonstrate that there is a significant and positive relationship between educational attainment and medication literacy, stating that the more education an individual has, the more educated and information-seeking individual that person is (30). Additionally, the lower medication literacy of the smoking and alcohol-consuming population, as noted in the study, is consistent with previous studies, and is the result of having lower health-related thinking (or having health-related thinking of a lower order) with a significant tendency to underestimate self-health-damaging risks, and therefore less self-controlled health-related thinking (31). These findings are inseparably related to the mechanisms of the effects of risk perception and low health-related thinking, or health-related thinking of a lower order, on one’s own behavior, and are inextrically related to low health literacy as a consequence of the manifested deficient health-related thinking.

**Figure 2:**
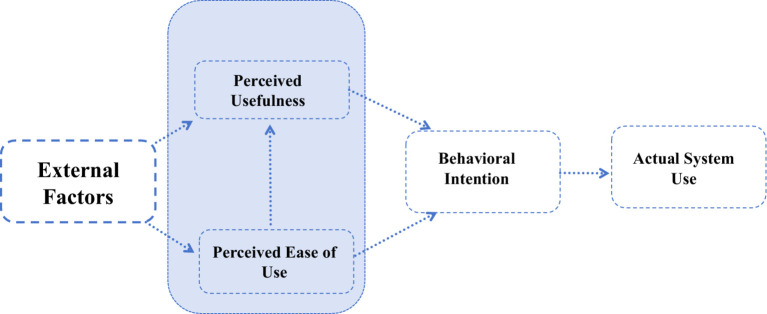
Technology acceptance model, TAM.

**Figure 3:**
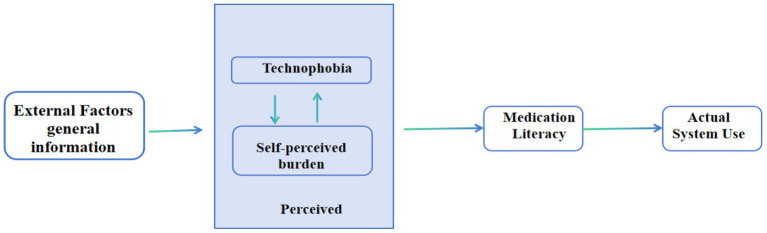
Conceptual framework diagram of this study.

The Technology Acceptance Model (TAM) is a framework that is widely used in information systems research to understand technology adoption (32, 37). It is based on perceived ease of use and perceived usefulness as the principal constructs for acceptance of technology (33) see Figures 2, 3. These two constructs are interrelated, and move users from acceptance of a technology to advanced dependency on the technology (34).

This research suggest that four primary interrelated fields: Information acquisition ability, Knowledge of medications, Communication and interactive abilities, and Critical thinking ability, are strongly positively related (*r* = 0.810–0.900). This supports the argument for the integrated structure of pharmacological literacy and demonstrates the emerging aggregative effect of “perceived usefulness (PU)” in the Technology Acceptance Model (TAM). This suggests that patients who perceive the usefulness of the digital technologies they are using demonstrate an improvement in their ability to retrieve, interpret, and make judgments regarding the information they have in a way that achieves “competency resonance.” This research suggests that technophobia and self-perceived burden are initial psychosocial impediments that “interfere” with people developing perceived ease of use and perceived usefulness of the system. This suggests that overcoming these barriers would lead to improved intentions and therefore sustained engagement with the technologies and, in turn, improved pharmacological literacy. Magi’s study demonstrates that health literacy impacts chronic illness self-care through medication adherence (35, 36); our study findings support this. Better health literacy results in a better self-care practice; hence, self-care practice at a higher health literacy level indicates improved adherence to instructions suggesting less or no accidental medication intake. The study found that patients with high-level medication literacy, who reported improved financial practices, self-problem, and self-care construct practices adhering to treatment and medication regimen, leading to improvedIndividual health outcomes (15). This study indicates that the health literacy levels among the older adults individuals having chronic disease comorbidities have low medication literacy. This is a facilitatory barrier to improved health outcomes. Hence, health practitioners need to develop age-sensitive measures to factor the inequities that occur in health literacy among the older adults. With the implementation of age-appropriate specific intervention programs, the older adults may understand at a conceptual level the tangible and intangible assets of better improved medication literacy, and consequently the health outcomes of the patients will be improved.

### Limitation

This study has several limitations that should be acknowledged when interpreting the results and when planning further studies. First, the study design used a cross-sectional survey method and was executed in one city, Shizuishan City, Ningxia, with a small and short survey. These factors constitute geographical and generalizability limitations for the study. Considering the differences in medical resources, health literacy, and lifestyle in the various regions, a single-center sample has the potential to be non-representative of the geriatric population with chronic comorbid conditions. Because of this, the study could be homogeneous in representing the results, and as such, is limited owing to the context of the study, this may lead to a homogeneity bias in the sample regarding socioeconomic status, healthcare policy environment, and cultural background, thereby partially limiting the external validity and generalizability of the research findings. Future studies should expand the sampling scope and conduct multicenter, cross-regional investigations to validate the broad applicability and stability of the model developed in this study across different healthcare settings. In the future studies, multi-centered and large survey samples should be used to evaluate the findings that have been collected. The other caveat would be that the data for this study was collected using a single cross-sectional method, which implies that there was no account of the subjects for different time periods, resulting in no longitudinal implications. A longitudinal method of study would allow for the variables to be measured at more than one time interval. Additionally, this method is a better control of variables to account for differences of the sample population because of the timing of the study with relation to the significant factors that are imperative. This will allow for the explanation of the phenomena, and clarify the key variables for the study.

Finally, while a number of major influencing factors have been elucidated in this study, scientifically rigorous intervention studies are still necessary once specific mechanisms have been identified. Healthcare professionals can develop individualized interventions relevant to each of the factors in which targeted health education and prescribing for different patient populations.

## Conclusion

According to LPA, the medication literacy characteristics of older adults patients with chronic comorbidities were categorized into four types: Medium Medication Literacy -low critical ability, Low Medication Literacy, High-Medication Literacy, and Medium Medication Literacy-High critical ability. Among the various categories of chronic senile comorbidities, differences were observed in factors such as drinking history, educational level, marital status, occupational status, personal monthly income, family location, caregiver availability, lifestyle, type of medical insurance, duration of daily exercise, duration of illness, number of hospitalizations in the past year, self-rated sleep status, age, self-perceived burden, and technophobia. The results of this study showed that there was a highly significant positive correlation between information acquisition ability, drug knowledge reserve, communication and interaction skills, critical thinking ability, and medication literacy was significantly positively correlated with self-perceived burden and technophobia. This study can assist healthcare professionals in identifying the factors that influence patients’ medication literacy abilities, enabling the implementation of targeted nursing programs aimed at improving treatment outcomes and the quality of life for this population.

## Data Availability

The datasets generated during the current study are not publicly available due to ongoing analyses for related research projects. Data are available from the corresponding author upon reasonable request (email: y18548161566@163.com).
